# Antioxidant Effect of a Marine Oligopeptide Preparation from Chum Salmon (*Oncorhynchus keta*) by Enzymatic Hydrolysis in Radiation Injured Mice

**DOI:** 10.3390/md9112304

**Published:** 2011-11-10

**Authors:** Ruiyue Yang, Junbo Wang, Zhigang Liu, Xinrong Pei, Xiaolong Han, Yong Li

**Affiliations:** 1 Department of Nutrition and Food Hygiene, School of Public Health, Peking University, Beijing 100191, China; E-Mails: ruiyue_yang@yahoo.com.cn (R.Y.); bmuwjbxy@bjmu.edu.cn (J.W.); zhigangliu718@gmail.com (Z.L.); rongpx@163.com (X.P.); hanxiaolong2005@tom.com (X.H.); 2 The Key Laboratory of Geriatrics, Beijing Hospital & Beijing Institute of Geriatrics, Ministry of Health, Beijing 100730, China

**Keywords:** bioactive peptide, GSH-Px, MDA, radioprotective, SOD

## Abstract

Marine oligopeptide preparation (MOP) obtained from Chum Salmon (*Oncorhynchus keta*) by the method of enzymatic hydrolysis, has been found to possess a radioprotective property through stimulation of the radiation-induced immunosuppression. The current study aimed to further investigate the free radicals scavenging and antioxidant effects of MOP in radiation injured mice. Female ICR mice (6–8 weeks old) were randomly divided into 5 groups, *i.e.*, blank control, irradiation control and MOP (0.225, 0.450 and 1.350 g/kg body weight) plus an irradiation-treated group. The result revealed that MOP significantly increased the white blood cell counts after irradiation, and lessened the radiation-induced oxidative damage. These effects may be caused by augmentation of the activities of antioxidant enzymes, such as SOD and GSH-Px, reduction of the lipid peroxidation (MDA level) in liver, and protection against radiation-induced apoptosis. Therefore, we propose that MOP be used as an ideal antioxidant to alleviate radiation-induced oxidation damage in cancer patients.

## 1. Introduction

In recent years it has been increasingly acknowledged that dietary proteins provide a rich source of bioactive peptides that can promote human health by reducing the risk of chronic diseases [1–3]. Similar to various endogenous bioactive peptides, such as some hormones, bioactive peptides from dietary sources have been defined as specific protein fragments that have a positive impact on body functions or conditions, and that may eventually benefit health. The activity is based on their inherent amino acid composition and sequence. The size of active sequences may vary from three to twenty amino acid residues, and many peptides are known to reveal multifunctional properties, such as antihypertension, immunomodulatory, antithrombotic, antioxidant, anticancer and antimicrobial activities, in addition to nutrient utilization [4].

Fish skin, bones, scales and residual minced meat, the by-products of the fish-processing industry, which usually cause wastage and pollution, can actually be turned into a high-protein food [5,6]. It has been found that enzymatic hydrolysis of dietary proteins offers a rapid and reproducible method for producing considerable bioactive peptide fractions, which are very likely to become health-beneficial food ingredients or nutraceutical preparations [7]. Actually, by enzymatic hydrolysis, bioactive peptides isolated from various kinds of fish, such as salmon [8,9], cod [10], yellow stripe trevally [11] and yellowfin sole [12], *etc*., have been proved to possess numerous biological activities beneficial for health. Therefore, we decided to conduct a research into enzymatic hydrolysis of Chum Salmon (*Oncorhynchus keta*), one of the most common species of fish in the Chinese farming industry, hoping that the findings of our study would be helpful in promoting the development of nutritional-value-added by-products, and alleviating the problem of fishing waste disposal.

Although being viewed as a useful remedy for cancer therapy, ionizing radiation can also cause a series of deleterious side effects, including oxidation damage and disorders of the immune system and the hematopoietic system. Our earlier research demonstrated that marine oligopeptide preparation (MOP), compounds of low molecule peptides extracted from salmon minced meat by enzymatic hydrolysis, possess a radioprotective property through stimulation of the radiation-induced immunosuppression, and may have a supplementary protective effect in cancer therapy [13]. The present study reported here mainly focuses on the free radicals scavenging and antioxidant effects of MOP in radiation injured mice.

## 2. Results and Discussion

### 2.1. Characterization of MOP

After HPLC purity and MALDI-TOF-MS analysis, we found that the molecular weight distribution of MOP was between 100 and 860 Da, and most of the peptides were distributed between 300 and 860 Da (85.86% of the total). Noting that the amount of free amino acids was 2.05%, it was deduced that the main composition of MOP was due to small peptides. The composition of amino acids was further analyzed. Generally, MOP was mainly composed of Glu > Asp > Lys > Leu > Arg > Gly, and the amounts of indispensable amino acids (IAAs) were approximately comparable to the amounts of dispensable amino acids (DAAs) (Table 1). The composition of amino acid was similar to that in other research in which soluble and fish protein hydrolysate contained high levels of Glu, Asp, Gly and Lys, whereas soy protein and casein contained high levels of Gln, Asn, Pro, Arg and Leu [14].

### 2.2. Effect of MOP on Irradiation-Reduced White Blood Cells Count

**Figure 1:**
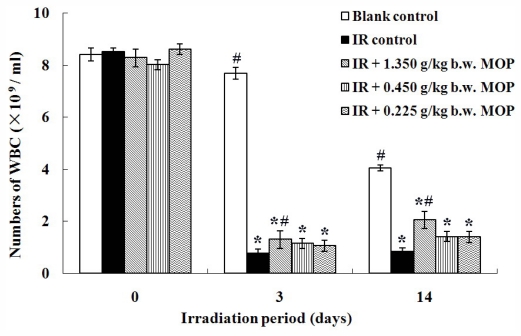
Effect of MOP on irradiation-reduced white blood cells count in mice after WBI (4.5 Gy). Values represented the mean ± S.D. (*n* = 10 per group). * *p* < 0.05 *versus* blank control; # *p* < 0.05 *versus* irradiation control. MOP, marine oligopeptide preparation; WBI, whole-body irradiation; S.D., standard deviation; IR, irradiation; b.w., body weight.

After radiation, hematological system usually displays morphological changes earliest. Many studies reported that the peripheral blood leukocyte count decreased significantly in mice after whole-body irradiation (WBI) [15,16]. As illustrated in Figure 1, the white-cell count 3 days following irradiation was (0.77 ± 0.116) × 10^9^/mL, significantly lower than in the non-irradiated controls (8.50 ± 0.709) × 10^9^/mL (*p* < 0.01). This indicates that irradiation-induced injury has seriously weakened intrinsic hematological system and immunomodulatory function. Oral administration with MOP markedly protected the mice from the irradiation-induced injury. On the 3rd day after being irradiated, the white cell count was (1.30 ± 0.337) × 10^9^/mL in high dose of MOP-treated animals, which were significantly higher than those of irradiated controls (*p* < 0.05). Similarly, on the 14th day after being irradiated, the white cell count in this group also increased significantly in comparison with irradiated controls (*p* < 0.05). In the 0.225 and 0.450 g/kg body weight (b.w.) MOP groups, the white cell counts were not significantly augment compared with the IR control group, although slight increases were observed. These results are consistent with our other data [13] in that WBI of mice do cause an exponential decline in cell survival of splenic cells and a depression capable of the response of splenic mononuclear cells to mitogens, both concanavalin A and lipopolysaccharide, and MOP can alleviate these inhibition effects. Considering these findings, we conclude that MOP possesses good irradiation protection property, which partly attributes to their strong immunostimulative activity.

### 2.3. Effect of MOP on Superoxide Dismutase (SOD) Activity in Serum and Liver, Glutathione Peroxidase (GSH-Px) Activity and Malondialdehyde (MDA) Level in Liver after WBI

**Figure 2:**
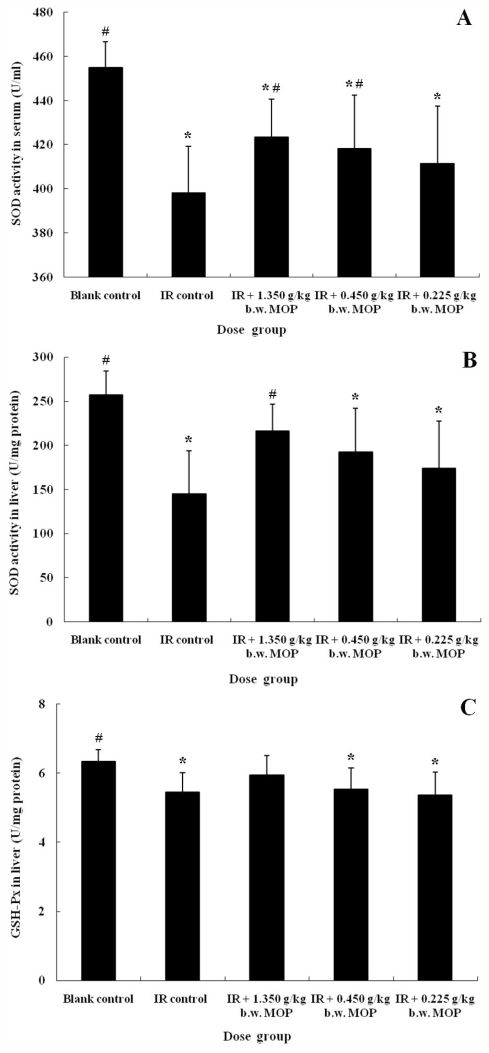

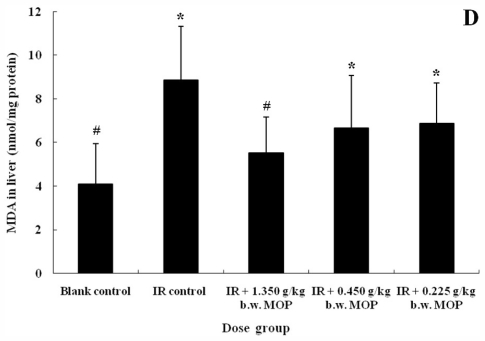
Effect of MOP on SOD activity in serum (**A**) and liver (**B**), GSH-Px activity (**C**) and MDA level (**D**) in liver after WBI (4.5 Gy). Values represented the mean ± S.D. (*n* = 10 per group). * *p* < 0.05 *versus* blank control; # *p* < 0.05 *versus* irradiation control. MOP, marine oligopeptide preparation; SOD, superoxide dismutase; GSH-Px, glutathione peroxidase; MDA, malondialdehyde; WBI, whole-body irradiation; S.D., standard deviation; IR, irradiation; b.w., body weight.

One of the major reasons for cellular injury after radiation exposure is the generation of free radicals and the increased levels of lipid peroxides in tissues and especially cell membranes, which are major determinants of cellular damage. Many investigators have reported the inhibition of antioxidant systems in blood and tissues of mice and rats, accompanied by an increase in lipid peroxide products after irradiation exposure [17–19]. Similar results were obtained in our current study. As shown in Figure 2, increased MDA level, decreased GSH-Px activity and SOD activity following irradiation were observed for 14 days after receiving 4.5 Gy irradiation. Products of lipid peroxidation such as MDA, have the ability to interact with and alter macromolecules, possibly resulting in diseases. Superoxide dismutase (SOD) and glutathione peroxidase (GSH-Px) constitute the enzymic antioxidant system, which scavenges oxidative stress (OS) production and lipid peroxidation. SOD is the only enzyme that disrupts superoxide radicals and is present in all cells with high amounts in erythrocytes. Besides, GSH-Px is an equally important antioxidant, which reacts with hydrogen peroxide thus preventing intracellular damage caused by the same. Thus, it has been demonstrated that the activity of antioxidative systems is suppressed after whole body γ-radiation exposure.

It is hypothesized that if the oxidative stress is involved in the origin of tissue damage, then successful antioxidant treatment should delay or prevent the onset of that damage [20]. Recently, there has been a great interest in the use of dietary agents as nontoxic antioxidants, compared with most synthetic radioprotectors, to reduce the deleterious side effects of radiation in cancer prevention and therapy [15,17,19]. In our study, compared with the irradiation control group, the inhibition of the MOP group was relieved. Treatment with 1.350 g/kg b.w. MOP, caused a significant increase of 6% (*P* < 0.05) and 49% (*P* < 0.05) in the SOD activity in serum and liver, respectively, in comparison with the irradiation control, as well as the activity in liver of 0.450 g/kg b.w. MOP group (*P* < 0.05) (Figure 2A,B). Although the elevated GSH-Px activity in liver of 1.350 g/kg b.w. MOP group was not significant, this activity recovered to the level of the blank control group (Figure 2C). Besides, the MDA level of 1.350 g/kg b.w. MOP group markedly decreased (*P* < 0.05) in comparison with IR control (Figure 2D). The increased activity of antioxidant enzymes in MOP-treated irradiated rats may be brought about either by facilitating the replacement of lost antioxidase activity in irradiated tissue, or by enhancing synthesis of essential repair enzymes. Thus, the finding suggests that MOP protects irradiation-induced injury partly by its strong free radical scavenging activity.

### 2.4. Effect of MOP on the Apoptosis Rate of Splenocytes after WBI

**Figure 3:**
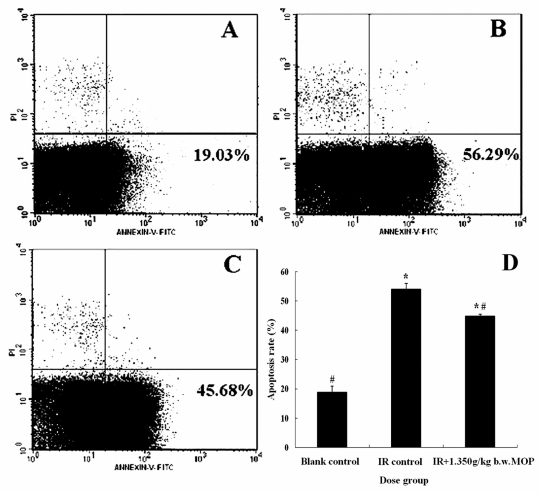
Effect of MOP on the apoptosis rate of splenocytes after WBI (6 Gy). (**A**) Blank control; (**B**) IR control; (**C**) IR + 1.350 g/kg b.w. MOP group; (**D**) Values represented the mean ± S.D. (*n* = 10 per group). * *p* < 0.05 *versus* blank control; # *p* < 0.05 *versus* irradiation control. MOP, marine oligopeptide preparation; WBI, whole-body irradiation; IR, irradiation; b.w., body weight; S.D., standard deviation.

Apart from oxidative stress, ionizing radiation have been shown to damage DNA, resulting in oxidative stress induced apoptosis [21] and various cancers [22,23]. The spleen is susceptible to ionizing radiation and is induced to undergo apoptosis on exposure to ionizing radiation. Several studies have shown that induction of apoptosis by WBI occurs in the cells of mice and rats [24,25]. The apoptosis of splenocytes in mice both with and without MOP treatment was evaluated by two-color flow cytometry (Annexin V-FITC/PI Staining) analysis. As shown in Figure 3, the apoptosis rate of splenocytes after irradiation was significantly higher than in the non-irradiated controls (*P* < 0.05), while MOP pretreatment lessened this tendency. In particular, compared with irradiation control, treatment with 1.350 g/kg b.w. MOP caused a decline of 18% in the apoptosis rate of splenocytes (*P* < 0.05). The result was similar to the findings of our other studies [13] in that MOP treated group significantly impaired the radiation-induced changes of levels of apoptosis-related proteins. Taken together, these results demonstrate that treatment with MOP protects the mitochondrial membrane against radiation-induced immune cell damage and genetic damage.

## 3. Experimental Section

### 3.1. Treatment of Mice with MOP

MOP was prepared from wild-caught Chum Salmon (*Oncorhynchus keta*) (average body weight, 1.47 kg) as described previously [26]. Six to eight weeks old female ICR mice, weighing 18–22 g, were obtained from the Animal Service of Health Science Center, Peking University. Animals were randomly divided into 5 groups, *i.e.*, blank control, irradiation control and 1.350, 0.45 and 0.225 g/kg b.w. MOP plus irradiation-treated group, with 10 animals in each group. Blank and irradiation control animals were fed AIN93M Diet from Vital River Limited Company (Beijing, China). The mice in the experimental group were fed 1.350%, 0.45% and 0.225% MOP in AIN93M diet (a dose of 1.350, 0.45 and 0.225 g/kg body weight (b.w.) MOP treatment in mice).

Their weight and food consumption were measured every week. No significant differences in diet or weight gain were found between the un-supplemented and supplemented mice (data not shown). All animals were adapted at animal colony facilities in the laboratory of animal service of Peking University for at least 1 week before treatment. Mice were housed four to five per cage. All animals were maintained at a constant temperature (23 ± 1 °C) and humidity (60 ± 10%) environment under a 12-h light/dark cycle (light on 07:30–19:30 h) with free access to food and water. Animal treatment and maintenance were carried out in accordance with the Principle of Laboratory Animal Care of the National Institutes of Health (NIH) [27] and the guidelines of the Peking University Animal Research Committee.

### 3.2. Irradiation of Animals with ^60^Co γ-Rays

Each mouse was placed individually in a close-fitting Perspex box (3 × 3 × 11 cm) and exposed to WBI with a ^60^Co source irradiator (Theratron-780 Teletherapy Unit, Health Science Center, Peking University, China). Briefly, mice placed in the box were exposed to WBI with a dose rate of 1.5 Gy min^−1^ for 4 min and source-surface distance of 150 cm.

Except for the blank control, mice of the other four groups were fed different diets for 14 days and received a single dose of WBI with 4.5 Gy of ^60^Co γ-rays on the 15th day. On the 3rd day and 14th day after irradiation, the effects of MOP on white blood cell counts were measured. Besides, on the 14th day after irradiation, the activity of antioxidant system and oxidative products were assayed. Except for the blank control, mice of the other four groups were fed different diets for 30 days and received a single dose of WBI with 6 Gy of ^60^Co γ-rays on the 30th day. 24 h after irradiation, to ascertain the effects of MOP on apoptosis, the apoptosis rate of splenocytes was measured by Annexin V-FITC/PI Staining analysis

### 3.3. Number of White Blood Cells

After irradiation, all mice were fed original diets. 20 μL peripheral blood added to 0.38 mL 1% HCL were collected triple, before irradiation and on the 3rd and 14th day after irradiation, respectively. The number of white blood cells in the peripheral blood was determined using an automated hematology analyzer (Beckman Coulter Inc., USA).

### 3.4. Measurement of Antioxidative Systems

Fourteen days after irradiation, animals were deeply anesthetized by CO_2_ inhalation and sacrificed. The liver was promptly dissected and perfused with 50 mM (pH 7.4) ice-cold phosphate buffer saline solution (PBS). Then the tissue was homogenized in 1/5 (w/v) PBS containing a protease inhibitor cocktail (Sigma–Aldrich) with 10 strokes at 1200 rpm in a potter homogenizer. Homogenate was divided into two portions and one part was directly centrifuged at 8000 g for 10 min. Supernatant were used to determine MDA levels. The second part of the homogenate was sonicated four times for 30 s with 20 s intervals using a VWR Bronson Scientific sonicator, centrifuged at 5000 g for 10 min at 4 °C, and the supernatants were collected to determine antioxidative systems and oxidative products.

SOD activity was measured according to the method of Fridovich *et al.* [28]. This method employs xanthine and xanthine oxidase to generate superoxide radicals. The superoxide radicals react with p-iodonitrotetrazlium violet to form a red formazan dye that was measured at 550 nm. Assay medium contained 0.01 M phosphate buffer, 3-cyclohexilamino-1-propanesulfonicacid (CAPS) buffer solution (50 mM CAPS, 0.94 mM EDTA) with pH 10.2, solution of substrate (0.05 mM xanthine and 0.025 mM 2-(4-iodophenyl)-3-(4-nitrophenol)-5-phenyl tetrazolium chloride, INT) and 80 μL xanthine oxidase. SOD activity in liver was expressed as U/mg of protein, while this value in serum was expressed as U/mL.

Measurement of GSH-Px activity was based on the following principle: GSH-Px catalyzes the oxidation of glutathione by cumene hydroperoxide [29]. In the presence of glutathione reductase and nicotinamide adenine dinucleotide phosphate (NADPH), the oxidized glutathione is immediately converted to the reduced form with a concomitant oxidation of NADPH to NADP+. The decrease in absorbance at 340 nm is measured. The enzyme unit of GSH-Px is defined as the number of micromoles of reduced NADPH oxidized per minute at 37 °C by 1 mL of supernatants under standard assay conditions. GSH-Px activity was expressed as U/mg protein.

### 3.5. Measurement of Oxidative Products

The level of oxidative products, *i.e.*, MDA, in liver tissue was determined using the method of Uchiyama and Mihara [30]. Half a milliliter of homogenate was mixed with 3 mL of H_3_PO_4_ solution (1%, v/v) followed by addition of 1 mL of thiobarbituric acid solution (0.67%, w/v). Then the mixture was heated in a water bath for 60 min. The colored complex was extracted into *n*-butanol, and the absorption at 532 nm was measured using tetramethoxypropane as standard. MDA levels were expressed as nmol per mg of protein.

### 3.6. Apoptosis Rate Measurement

The apoptosis of spleen was evaluated by two-color flow cytometry (Annexin V-FITC/PI Staining) analysis. The spleens were removed using a sterile technique, and placed in sterile plates containing Hank’s balanced salt solution (HBSS, containing 137.93 mM NaCl, 5.33 mM KCl, 4.17 mM NaHCO_3_, 0.441 mM KH_2_PO_4_, 0.338 mM Na_2_HPO_4_ and 5.56 mM d-Glucose), then the splenocytes were dissociated from the connective tissue capsule by gently pressing the organ through a 200 mesh, sterile metal sieve. The sieve was rinsed with HBSS, and the suspension collected in sterile 15 mL conical tubes, and red blood cells in the cell suspension were dissolved in haemolyzed solution (7 g/L NH_4_Cl and 2.6 g/L Tris-HCl). The resulting single-cell suspension was then washed twice with HBSS and centrifuged (180× *g*, 10 min). The supernatant was discarded, and the cells were resuspended in 200 μL ice-cold binding buffer and 10 μL horseradish peroxidase (FITC)-labled Annexin V and 5 μL propidium iodide (PI) were added in. The cell suspension was gently mixed and incubated in the dark for 15 min at room temperature. Apoptosis was determined by flow cytometry (Becton Dickinson, USA). In the study Annexin V positive and PI negative cells were defined as apoptotic cells. Both Annexin V-FITC and PI negative cells were considered as viable cells, while both Annexin V-FITC and PI positive cells were considered as late apoptotic or already dead cells.

### 3.7. Statistical Analysis

The one-way analysis of variance (ANOVA) test and multiple comparison of Dunnett’s *t*-test were used to evaluate the differences of parametric samples between control and MOP groups, and data were expressed as mean ± standard deviation (SD) for the values from the number of experiments shown in the figures.

## 4. Conclusions

This current research demonstrates that MOP significantly increases the white blood cell counts after irradiation, and alleviates the radiation-induced oxidation damage. These effects may be caused by augmentation of the activities of antioxidant enzymes, such as SOD and GSH-Px, reduction of the lipid peroxidation, and protection against radiation-induced apoptosis. Therefore, we propose that MOP be used as an ideal antioxdant to alleviate radiation-induced oxidation damage in cancer patients. Further studies should be undertaken to separate and identify certain specific peptides in the mixture responsible for the activity.

## Figures and Tables

**Table 1:** Amino acid composition of marine oligopeptide preparation (MOP) from salmon.

Amino acid	No. residues/100 residues
Arginine	7.12
Histidine	3.39
Isoleucine	3.76
Leucine	7.71
Lysine	9.18
Methionine	3.52
Phenylalanine	4.46
Threonine	4.67
Tryptophan	0.19
Valine	5.17
Indispensable AA (IAA)	49.16
Alanine	5.70
Aspartic acid	10.45
Cystine	1.05
Glutamic acid	15.72
Glycine	6.56
Proline	3.61
Serine	4.10
Tyrosine	3.65
Dispensable AA (DAA)	50.84

MOP, marine oligopeptide preparation; AA, amino acid.
